# Supplementary material for: The causal role of circulating inflammatory markers in osteoporosis: a bidirectional Mendelian randomized study
Source: Front Immunol. 2024 Jul 18;15:1412298. doi: 10.3389/fimmu.2024.1412298 (PMC11291241; doi:10.3389/fimmu.2024.1412298)
Supplement: Supplementary file 1 [file DataSheet_1.zip › supplementary material/OSTEOPOROSIS-CIM/GCST90274836.tsv.gz_forest.pdf]

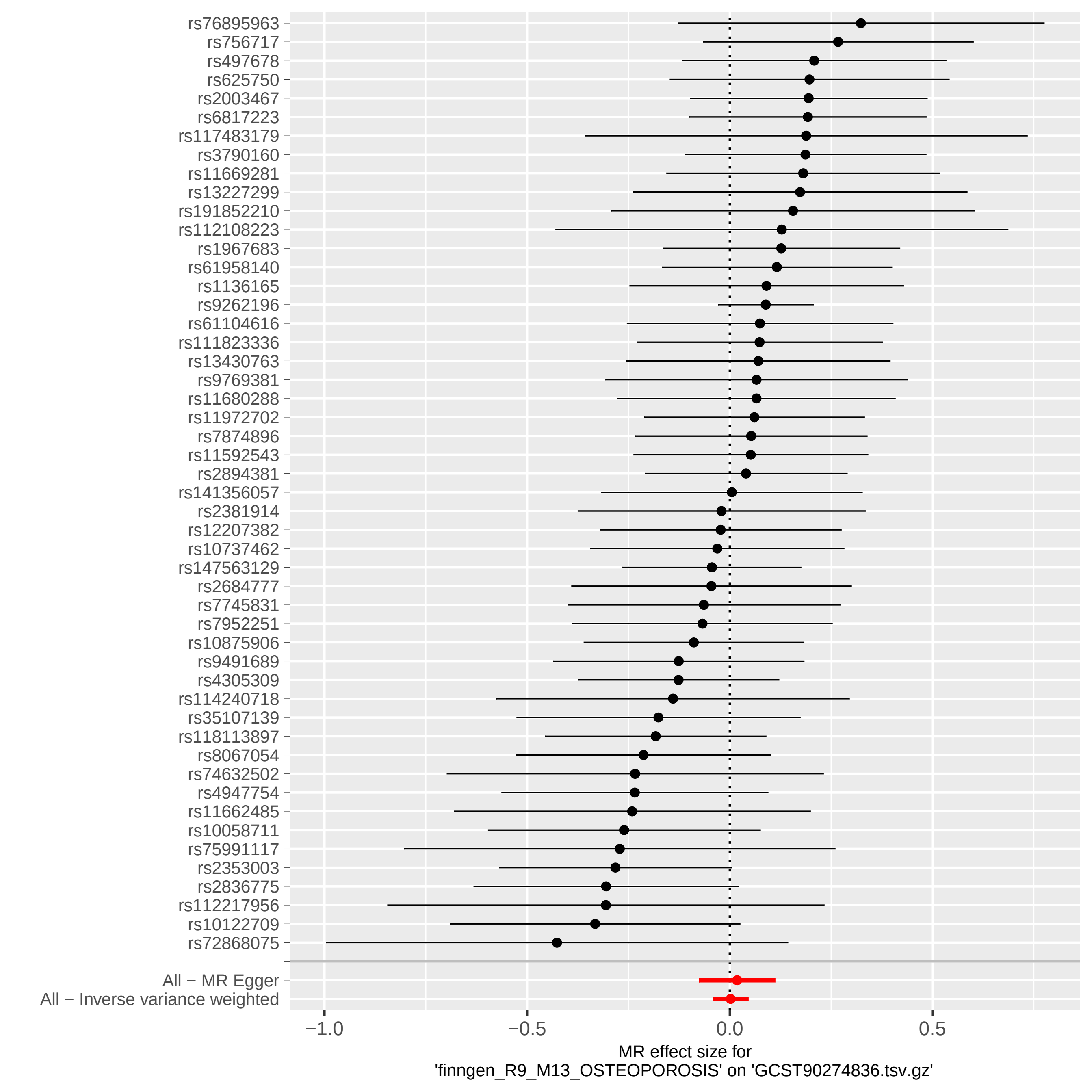
rs76895963
rs756717
rs497678
rs625750
rs2003467
rs6817223
rs117483179
rs3790160
rs11669281
rs13227299
rs191852210
rs112108223
rs1967683
rs61958140
rs1136165
rs9262196
rs61104616
rs111823336
rs13430763
rs9769381
rs11680288
rs11972702
rs7874896
rs11592543
rs2894381
rs141356057
rs2381914
rs12207382
rs10737462
rs147563129
rs2684777
rs7745831
rs7952251
rs10875906
rs9491689
rs4305309
rs114240718
rs35107139
rs118113897
rs8067054
rs74632502
rs4947754
rs11662485
rs10058711
rs75991117
rs2353003
rs2836775
rs112217956
rs10122709
rs72868075
All − MR Egger
All − Inverse variance weighted
−1.0
−0.5
0.0
0.5
MR effect size for
'finngen_R9_M13_OSTEOPOROSIS' on 'GCST90274836.tsv.gz'
